# Evaluation of the GeneXpert MTB/RIF to diagnose tuberculosis in a public health laboratory

**DOI:** 10.11606/s1518-8787.2024058005306

**Published:** 2024-02-09

**Authors:** Mohanna Damasceno Arbués, Maria Lúcia Rosa Rossetti

**Affiliations:** I Universidade Luterana do Brasil Programa de Doutorado em Biologia Molecular e Celular Aplicada à Saúde Canoas RS Brasil Universidade Luterana do Brasil. Programa de Doutorado em Biologia Molecular e Celular Aplicada à Saúde. Canoas, RS, Brasil

**Keywords:** Tuberculosis, Mycobacterium Tuberculosis, Clinical Laboratory Techniques

## Abstract

**OBJECTIVES:**

To evaluate the performance of geneXpert MTB/Rif versus conventional methods (bacilloscopy and culture) in the diagnosis of tuberculosis in a Central Public Health Laboratory (LACEN, Tocantins), Northern Brazil.

**METHODS:**

Retrospective study, with information from 1,973 suspected cases of tuberculosis from patients treated from January 2015 to December 2020.

**RESULTS:**

From the culture (reference standard), the sensitivity, specificity, positive predictive value, negative predictive value, and accuracy of the geneXpert MTB/Rif were 100%, 97%, 74%, 100%, and 97%, respectively, against 85%, 98%, 80%, 98%, and 97% of bacilloscopy.

**CONCLUSIONS:**

The geneXpert MTB/Rif performed similarly to culture and better than bacilloscopy. Although positive cases with negative culture should be evaluated with caution, its routine use is important for the early detection of tuberculosis.

## INTRODUCTION

Tuberculosis is an infectious disease with high morbidity and mortality rates worldwide^1^. It is caused by *Mycobacterium tuberculosis* (MTB) and can cause pulmonary and extrapulmonary infections^2^. Globally, it is estimated that, in 2020, tuberculosis affected approximately 9.9 million people, accounting for 1.3 million deaths^3^. In Brazil, approximately 70,000 new cases of tuberculosis were reported in 2021,32 cases per 100,000 inhabitants, whereas the number of deaths in 2020 was 4,543 (2.1 deaths per 100,000 inhabitants)^1^.

Tocantins is a state in the northern region of Brazil and its capital Palmas is the youngest and least populous in the country. Although the tuberculosis incidence rate (13.9 per 100,000 inhabitants) is lower than the national rate (32.1 per 100,000 inhabitants), it has been increasing. In 2021, the number of confirmed cases jumped from 213 (in 2020) to 283, and the same was registered in its capital, which had practically doubled the number of confirmed cases in the same period (from 34 to 67 cases)^4^. Accurate and rapid diagnosis is essential for epidemiological control of the disease and for a better prognosis.

In Brazil, since 2013, the rapid molecular test (RMT) geneXpert MTB/Rif (Cepheid, Sunnyvale, CA, USA), a real-time PCR (qPCR), which detects *M. tuberculosis* DNA and resistance to the antibiotic rifampicin^5^, with sensitivity ranging from 68% to 100% and specificity from 91.7% to 99.3%, has been introduced into the tuberculosis diagnostic routine in laboratories located in high prevalence regions^6^. Recently, a new version, geneXpert MTB/Rif Ultra, was introduced to improve the sensitivity of the method. Assessments of this method’s contribution in less populated Brazilian regions, such as the North, are still scarce. Considering the increase in cases in the state of Tocantins, analyzing the use of this technology in tuberculosis diagnosis can contribute to actions aimed at controlling the disease.

This article aimed to evaluate the performance of the geneXpert MTB/Rif compared to conventional methods (bacilloscopy and culture) in the diagnosis of tuberculosis at a Central Public Health Laboratory in the State of Tocantins (LACEN-TO).

## METHODS

### Study Design and Sample

**Figure 1:**
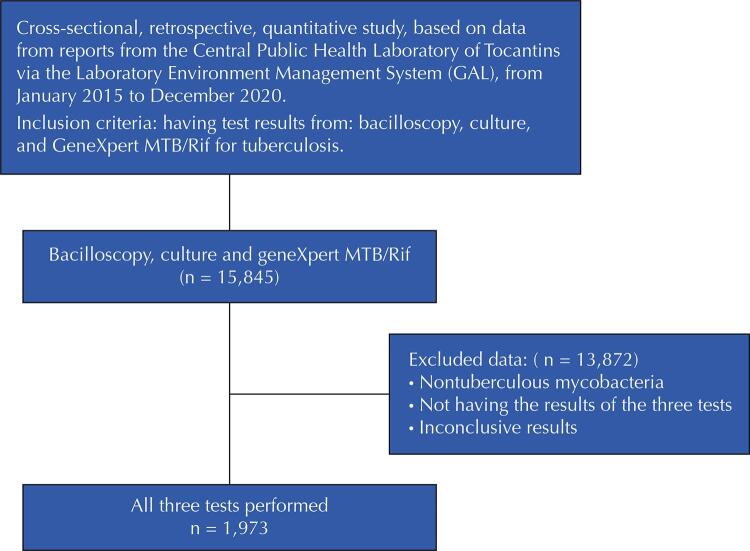
Flowchart of the study design.

LACEN-TO is a public reference state laboratory, responsible for the training, monitoring, supervision, evaluation, and quality control of the state laboratory network. Additionally, it is also responsible for the segment of cultures coming from the entire state of Tocantins and surrounding states, and is responsible for carrying out the geneXpert MTB/Rif and the antimicrobial sensitivity testing (AST) concomitantly, when necessary (Figure 1).

MTB: Mycobacterium tuberculosis.

#### Bacilloscopy/Ziehl Neelsen Staining

Samples from patients with suspected tuberculosis treated in the public health system were microscopically observed for the presence of acid-fast bacilli (AFB) using Ziehl Neelsen (ZN) staining, as recommended by the Brazilian Ministry of Health^1,7^.

#### Culture

When necessary, samples from suspected tuberculosis patients were previously decontaminated using the Petrof method and then inoculated on Lowenstein Jensen (LJ) medium, subsequently incubated at 37°C for 8 weeks. Cultures showing growth suggestive of *M. tuberculosis* were submitted to ZN bacilloscopy and immunochromatographic or biochemical tests to confirm the microorganism^1,7^.

#### Antimicrobial sensitivity testing (AST)

The positive isolates for *M. tuberculosis* were tested against the first-line drugs (isoniazid, rifampicin, streptomycin, and ethambutol), using the AST based on the proportion method in Löwestein-Jensen (LJ) medium, recommended by the Brazilian Ministry of Health^7^.

#### GeneXpert MTB/Rif

Molecular analyses were carried out using the inputs part of the Xpert® MTB/Rif assay, according to the manufacturer’s instructions, after decontaminating the sample in a diluent solution (2:1 ratio, mixture of NaOH and isopropanol)^8^.

## Statistical Analysis

All the data obtained were entered and tabulated in Microsoft Excel spreadsheets, compiled and analyzed using SPSS® software (version 23.0, Chicago, USA, Statistical Package for the Social Sciences). Assessments of possible statistical differences between qualitative variables were verified by Pearson’s chi-square test or Fisher’s exact test, as appropriate. Comparisons of the analytical performances between the gold standard (culture) and the bacilloscopy and RMT tests were estimated by sensitivity, specificity, positive predictive value (PPV), negative predictive value (NPV), and accuracy with the respective 95% confidence intervals (95%CI). All analyses were bilateral with pre-established significance level for alpha error of 5% *(*p ≤ 0.05).

## Ethical Aspects

This study was approved by the research ethics committee of the Universidade Luterana do Brasil (Protocol number: 4.739.801, May 21, 2021).

## RESULTS

A total of 1,973 tuberculosis test results were evaluated. Regarding the evaluated diagnostic techniques for tuberculosis, the positivity of the samples was 143 (7.2%) for bacilloscopy, 153 (7.8%) for culture, and 206 (10.4%) for geneXpert MTB/Rif. Of these geneXpert MTB/Rif evaluated for rifampicin resistance, only 03 (1.5%) samples showed resistance, and only a single sample was confirmed in AST.

Correlating the demographic variables with the diagnostic techniques , there was no statistically relevant association (p ≤ 0.05) between sex and the bacilloscopy technique (Table 1). The positivity of the samples, regardless of the technique employed, was higher in males and in the age group of 21 to 40 years. Regarding indigenous ethnicity, there was an average percentage of positivity of 8.7% among the different tuberculosis diagnostic techniques (Table 1).

**Table 1:** Sociodemographic characteristics of patients with suspected tuberculosis by different diagnostic techniques.

Characteristic	Bacilloscopy			Culture			GeneXpert MTB/Rif		
		
	Positive	Negative	p-value^a^	Positive	Negative	p-value^a^	Positive	Negative	p-value^a^
					
	n (%)	n (%)		n (%)	n (%)		n (%)	n (%)	
Sex			0.19			0.03			0.02
Male	91 (63.6)	1,064 (58.1)		102 (66.7)	1,053 (57.9)		135 (65.9)	1,020 (57.7)	
Female	52 (36.4)	766 (41.9)		51 (33.3)	767 (41.1)		70 (34.1)	748 (42.3)	
Age range			0.01			0.01			0.01
0–20	14 (9.8)	163 (8.9)		11 (7.2)	166 (9.1)		20 (9.8)	157 (8.9)	
21–40	62 (43.4)	617 (33.7)		69 (45.1)	610 (33.5)		85 (41.5)	594 (33.6)	
41–60	45 (31.5)	536 (29.3)		47 (30.7)	534 (29.3)		64 (31.2)	517 (29.2)	
61–80	19 (13.3)	437 (23.9)		22 (14.4)	434 (23.8)		32 (15.6)	424 (24)	
81–100	3 (2.1)	77 (4.2)		4 (2.6)	76 (4.2)		4 (2)	76 (4.3)	
Ethnicity			< 0.01			0.02			< 0.01
Indigenous	13 (9.1)	64 (3.5)		11 (7.2)	66 (3.6)		20 (9.8)	57 (3.2)	
Other	130 (90.9)	1,766 (96.5)		142 (92.8)	1,754 (96.4)		185 (90.2)	1,711 (96.8)	

MTB: *Mycobacterium tuberculosis.*

^a^ p-value: p ≤ 0.05.

When the geneXpert MTB/Rif and culture results were compared (Table 2), the geneXpert MTB/Rif detected *M. tuberculosis* in all 153 specimens that were also culture positive, with a sensitivity of 100%. However, the specificity was 97% due to the geneXpert MTB/Rif being positive in 53 samples that were culture negative. Of these, 15 were also positive in bacilloscopy and, specifically in this test, the vast majority had a count of less than one bacillus, and, therefore, a low amount of bacillary bacteria in the smear. The other 38 were positive only in the geneXpert MTB/Rif.

**Table 2:** Comparison of the analytical parameters of the different tuberculosis diagnostic techniques.

Characteristic	Culture			Sensitivity	Specificity	PPV	NPV	Accuracy

	Positive	Negative	p-value^a^					
						
	n (%)	n (%)		% (95%CI)	% (95%CI)	% (95%CI)	% (95%CI)	% (95%CI)
GeneXpert MTB/Rif								
Positive n (%)	153 (100)	53 (2.9)	< 0.01	100 (97.6–100.0)	97.1 (96.2–97.8)	74.7 (68.9–79.0)	100 (99.6–100.0)	97.3 (96.5–97.9)
Negative n (%)	0 (0)	1,767 (97.1)						
Bacilloscopy								
Positive n (%)	122 (80.3)	21 (1.2)	< 0.01	85.3 (78.4–90.7)	98.4 (97.7–99.0)	80.3 (73.9–85.4)	98.8 (98.3–99.2)	97.4 (6.6–98.1)
Negative n (%)	30 (19.7)	1,800 (98.8)						
GeneXpert MTB/Rif								
Baciloscopy				96.5 (92.0–98.9)	96.2 (92.2–97.0)	66.7 (61.3–71.6)	99.7 (99.3–99.9)	96.2 (95.3–97.0)
Positive n (%)	138 (66.7)	5 (0.3)	< 0.01					
Negative n (%)	69 (33.3)	1,761 (99.7)						

MTB: *Mycobacterium tuberculosis*; PPV: positive predictive value; NPV: negative predictive value; 95%CI: 95% confidence interval.

^a^ p-value: p ≤ 0.05.

The sensitivity and specificity of bacilloscopy compared to culture was 85.3% and 98.4%, respectively, with PPV and NPV of 80.3% and 98.8% (Table 2).

**Figure 2:**
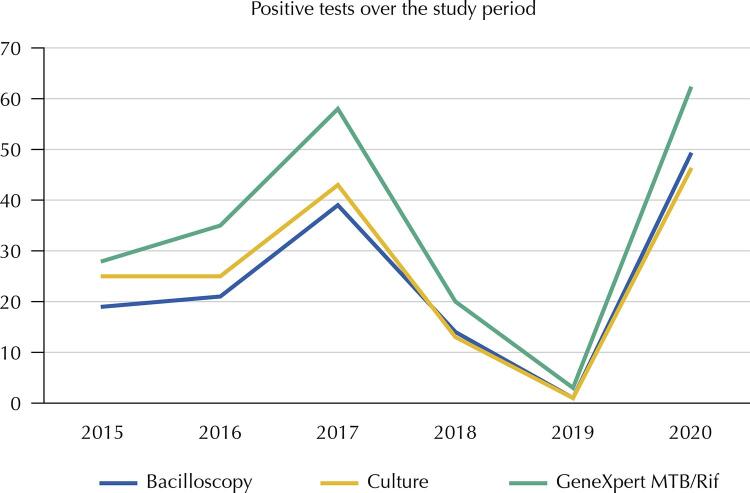
Positive tests for tuberculosis by different diagnostic techniques from 2015 to 2020.

When looking only at the positive results of the different tuberculosis diagnostic techniques evaluated during the study period, it was found that there was a variation in the number of diagnoses over the years, being higher in 2017 (approximately 46 tests on average) and 2020 (approximately 52 tests on average) regardless of the technique used. However, it is clear that the geneXpert MTB/Rif detected more tuberculosis cases throughout the period. In 2020, this detection increased even more, by around 10% when compared to 2017 (increased from 59 to 62 positive results), the previous year with the highest detection of this technique (Figure 2).

MTB: Mycobacterium tuberculosis.

## DISCUSSION

The difficulties encountered in laboratory techniques for detecting *M. tuberculosis* mean that new methodologies to improve the diagnosis of tuberculosis are constantly being sought.

In this work, the performance of the geneXpert MTB/Rif for the diagnosis of tuberculosis was analyzed against the most used techniques, bacilloscopy and culture, in a central public health laboratory located in the northern region of Brazil, in the state of Tocantins, where there are few related studies and, in recent years, there has been an increase in the number of cases.

When comparing the results of the diagnostic tests for tuberculosis, there was greater positivity obtained with the geneXpert MTB/Rif compared to the other techniques. The test detected *M. tuberculosis* in 63 more samples than bacilloscopy and in 53 more samples than culture. This may have been due to the ability of the geneXpert MTB/Rif to detect DNA by real-time PCR, with a detection limit of 131 CFU/ml of sample^9^. Additionally, the test has the ability to detect unviable bacilli, which culture does not^10^and, in the case of bacilloscopy, around 5,000 to 10,000 bacilli/ml of sample are required for a positive result^11^. It is also worth mentioning that decontaminating the samples before sowing the culture can make part of the bacilli unviable, due to the alkalinization or acidification of the medium^12^, which, in turn, does not interfere with the geneXpert MTB/Rif^13^. However, it is also important to consider the possibility of false positives, as the test can detect minute amounts of DNA from different sources, including patients who have previously had tuberculosis^14^.

Regarding the detection of rifampicin resistance by the geneXpert MTB/Rif, of the three resistant *M. tuberculosis* isolates, only one was also resistant in the AST, which, in addition to the difficulties already mentioned with the culture, could be false positives, since the geneXpert MTB/Rif can detect silent mutations in the rpoB gene, or it could also be mixed infections, in which there are susceptible and resistant *M. tuberculosis* isolates in the same sample^15,16^.

Regarding the 203 *M. tuberculosis* samples detected as sensitive in the geneXpert MTB/Rif, it was possible to confirm 58, since they were the only samples with AST results, which was a limitation of this study. However, even with this limitation, the 1.5% rate of rifampicin resistance found by the geneXpert MTB/Rif was similar (2%) to the study carried out in Rio de Janeiro by Sieiro et al.^17^.

Observing the sociodemographic profile of the sample studied, a representation very close to the Brazilian reality was verified, where the males and the age group of 21–40 years were the most affected by the disease, according to the Tuberculosis Epidemiological Bulletin^4^. However, the percentage of the indigenous ethnic group affected by the disease was higher (8.7%) than that presented in the Bulletin (2.1%). Nevertheless, the tuberculosis positivity defined by the MTB/Rif geneXpert in our study was 10.7%, on average, in the period studied and that presented by the Tuberculosis Epidemiological Bulletin was 12.9%^4^. Notably, statistical significance was found in most of the correlations when sociodemographic data were evaluated with the different tuberculosis diagnostic techniques. This is explained by the large sample size of the study, which is generally not the case in other studies.

In this study, it was also observed that the performance of the geneXpert MTB/Rif in diagnosing tuberculosis was excellent, when compared to culture (gold standard), with high levels of sensitivity, specificity, and accuracy (100%, 97%, and 97% respectively), being superior to the performance parameters obtained by Elbrolosay et al.^18^, in a study in Egypt, which were 90% and 87% of sensitivity and specificity, respectively. Studies in Saudi Arabia and India showed the same sensitivity as this study for the geneXpert MTB/Rif technique^19,20^. A study carried out in Brazil by Brito et al.^21^, in Rio de Janeiro, one of the cities with the highest incidence of tuberculosis, presented a lower sensitivity of 82% and similar specificity of 96%. Another study, in Goiás (Brazil), conducted by Dietz et al.^22^ reported an 87% sensitivity and 97% specificity. However, it is important to emphasize the relevance of our study due to the high sample number (n = 1,973), since most studies of this nature have relatively small samples.

When bacilloscopy was compared with culture, the sensitivity and specificity were of 85% and 98%, respectively. However, five bacilloscopy-positive samples were negative for the geneXpert MTB/Rif and in culture. These could be nontuberculous mycobacteria (NTMs) that are not detected in the geneXpert MTB/Rif. However, as the NTMs would also be identified in the culture, a possible inhibition of the PCR by substances present in the extracted DNA could be considered^23^. The sensitivity of bacilloscopy in this study was higher than the sensitivity verified in a study in Thailand (60.5%)^26^. In the study by Brito et al.^21^, in Brazil, the sensitivity was also lower (68%), and specificity was 96% compared to 98% in this study. Therefore, there is a possibility of false negatives when bacilloscopy is the only technique used for diagnosing tuberculosis, which occurs with a certain frequency, since culture is a time-consuming method for obtaining the results and ends up having only an epidemiological purpose and the possibility of defining a resistance profile by the AST, making it necessary to associate or replace the technique^27^.

In this study, the PPV of the geneXpert MTB/Rif was 74%, lower than that found in bacilloscopy (80%). The NPV was 100% and the bacilloscopy 98%. In a study by Brito et al.^21^ in Brazil, the NPV for the Xpert MTB/Rif gene was superior to that of bacilloscopy (94% and 87%, respectively). In the review study by Faria et al.^6^, the PPV for the geneXpert MTB/Rif ranged from 79% to 96% and the NPV from 84% to 99%. The high NPV of the geneXpert MTB/Rif showed the possibility of using the test to exclude the disease, but, on the other hand, its slightly lower PPV warns of the possibility of false positive results due to its high sensitivity^28,29^.

It is important to emphasize that throughout the study period, the geneXpert MTB/Rif was the test that detected the most cases of tuberculosis. In 2020, the last year evaluated, an even greater increase (around 10%) in positivity was observed, when compared to the other techniques. This may have been due to the implementation of the geneXpert MTB/Rif Ultra version, which was introduced to improve the sensitivity of the method. However, further studies are needed to evaluate the performance of this new version as, according to the study by Dorman et al.^30^, there was an actual increase in sensitivity, but at the expense of methodological specificity, which may not be interesting in certain situations for diagnosing tuberculosis.

The findings of this study indicated that the geneXpert MTB/Rif performed better than bacilloscopy and similarly to culture, which makes it a valuable tool in the diagnosis of tuberculosis, since its rapid results associated with clinical findings enable assertive therapeutic decisions to contain the progression of the disease and its spread, contributing to the clinical and epidemiological situation of tuberculosis. Future studies on the performance of the geneXpert MTB/Rif in relation to the detection of rifampicin resistance and the improved detection capacity of *M. tuberculosis* by the ultra version should be carried out to better elucidate the singular features of this promising test.
